# Respiratory Disorders among Dust Exposed Workers

**DOI:** 10.31729/jnma.3987

**Published:** 2019-02-28

**Authors:** Suman Bahadur Singh, Swotantra Gautam, Narendra Bhatta, Gambhir Shrestha, Rabin Gautam, Sagar Poudel

**Affiliations:** 1 B P Koirala Institute of Health Sciences, Dharan, Nepal; 2 Department of Cancer Prevention, Control and Research, B.P. Koirala Memorial Cancer Hospital, Bharatpur, Nepal; 3 HERD International, Thapathali, Kathmandu, Nepal; 4 All India Institute of Medical Sciences, New-Delhi, India

**Keywords:** *dust exposure*, *peak expiratory flow rate*, *respiratory disorder*

## Abstract

**Introduction:**

Exposure to dusts and hard physical work is common in developing industrialized countries. Acute and chronic respiratory illnesses are highly been reported from jute and textile industry. This study was undertaken to explore status of respiratory health among the workers of jute and textile industries.

**Methods:**

This descriptive cross-sectional study enrolled 315 workers from each of textile and jute industry of Eastern Nepal. Almost all the workers were selected from the textile industry whereas those from dust prone areas of jute industry. Workers were interviewed using pre-tested questionnaires. Measurement of height, weight and peak expiratory flow rate was done.

**Results:**

Majority were non-smokers in both the industries 230 (73%) in Jute vs. 223 (70.8%) in Textile. Most of the workers had the working experience of less than five years; jute 134 (42.5%) vs. textile 180 (57.1%). Upper respiratory disorder was found in more than 1/5 of workers (68) in jute vs. 1/20 of workers (18) in textile industry. One and two workers suffered from chronic bronchitis in the jute and the textile industry respectively. Chest tightness was reported among 4 (1.3%) in jute vs. 17 (5.4%) in textile workers, cough symptoms among 86 (27.3%) in jute vs. 26 (8.3%) in textile industry. Low practice of personal protective measure was seen in both industries. The mean score of PEFR of workers in jute mill was lower than the workers in textile industry.

**Conclusions:**

Workers with acute respiratory disorders were more in the jute industry while chest tightness was more in the textile industry. Chronic respiratory problems did not appear to be alarming in both the industries. Use of personal protective measures should be promoted among the dust exposed workers.

## INTRODUCTION

Exposure to high levels of dusts among industrial workers is common in developing and newly industrialized countries.^1^ Pulmonary response to hazardous airborne particles at workplaces produces airway disorders.^2^ Occupational exposures to airborne particulates is estimated to cause 12% of deaths due to chronic obstructive pulmonary disease and occupational health is of major concern in the South-East Asia Region.^3,4^

Cough, expectoration, breathlessness, headache and nasal blockage were found to be the main complaints among workers in jute mill and was suspected in workers with more than six years employment in textile industry.^5,6^ Studies have revealed Peak Expiratory Rate (PEFR) significantly lower in high dust exposure group in comparison to less exposure group.^7,8^ Current occupational safety, health and working conditions in Nepal are indicative of potential risks of health hazards at workplaces in the industrial establishments.^9^

We undertook this study to explore the status of respiratory problems among workers of jute and textile industry; to relate exposure to dust and PEFR.

## METHODS

The descriptive cross-sectional study was designed to enroll workers from textile industry and jute industry in Dharan-Biratnagar Industrial Corridor of Nepal from August 2014 to March 2015. Ethical Approval was obtained from Institutional Ethical Review committee of BPKIHS. The subjects who were engaged in the industries and not working as trainees and giving consent were included in the study. Unit of the study was an individual worker.

Prior studies have shown that respiratory problems among workers (25.3%) in textile industry is more thanamong workers (16%) in jute mill.^10,11^ Sample size was calculated by taking the lower prevalence of respiratory problems among workers in jute mill industry i.e. 16%.^10^

Sample Size was calculated by using the formula


$$\text{n}={\text{Z}}^{2}{\text{pq/e}}^{2}$$

where P is the prevalence of the reference study (p=16), q is the complement of p, i.e., q= 100-p, e is allowable error, which is taken to be 20% of p in this study and Z is the standard normal variate, which is 1.96 for 95% confidence interval. Hence, it gives us 525 sample size.

Adding 20% for non-responsive participants, the final sample size was 630 participants.

Equal number of participants was enrolled from the jute industry (315) and the textile industry (315). Almost all the workers were selected from the textile industry while the workers from the jute industry were selected from batching, spinning, weaving and drawing, which were supposed to have higher dust exposure. Consent was taken from authorities of the industries to conduct the study. Informed consent was taken from the participants.

Interview technique was applied to collect data by using pretested questionnaires. Measurement of height and weight was done. The weight of the workers was taken in minimum clothing and without shoes. Peak expiratory flow rate (PEFR) was measured by a vitalograph Peak expiratory flow meter. The purpose and technique of the test was explained to the study subjects followed by demonstrating the manoeuvre. The subjects were allowed to make two-practice attempts.^12^ An acceptable peak expiratory flow value was defined as the one that was produced with the hardest blow, after taking a maximum deep inhalation, as was interpreted on visual appearance. The highest of the three acceptable readings was recorded as the peak expiratory flow of the individual.

The predicted peak values of PEFR was calculated by the models for predicting peak expiratory flow rate (PEFR) which was developed for North Indian healthy population.^13^ Asthma was suspected for individual who's PEF was <80% predicated.^14^ Other features of asthma were enquired and suggested as per the need. Shortness of breath, wheeze and chest tightness were considered as lower respiratory tract symptoms. Chronic bronchitis was said when sputum production occurring on most (>5) days of a week for at least 3 months a year for at least 2 consecutive years. Chronic cough was said when cough without sputum for >5 days a week for at least 2 consecutive years. Dyspnea was said when having to walks lower than a person of the same age at an ordinary pace on a level ground because of breathlessness. Analysis of data was done using Statistical Package for Social Sciences (SPSS, version 16.0).

## RESULTS

A total of 630 participants working in various departments or units of jute and textile industry were enrolled in the study. Higher proportions of workers were males in both the industries, 269 (85.4%) workers in jute industry and 301 (95.6%) workers in textile industry respectively. Majority of the workers were married, 261 (82.9%) workers in jute industry and 243 (77.1%) workers in textile industry. About 53 (16.8)% and 42 (13.3%) workers were illiterate in the jute and textile industry respectively (Table 1).

**Table 1.** Socio-demographic characteristics of the workers in the jute and textile industry.

Characteristics	Jute Industry (n = 315) n (%)	Textile Industry (n=315) n (%)
Gender		
Male	269 (85.4)	301 (95.6)
Female	46 (14.6)	14 (4.4)
Marital status		
Unmarried	41 (13.0)	70 (22.2)
Married	261 (82.9)	243 (77.1)
Divorced/	5 (1.6)	1 (0.3)
Separated		
Widowed	8 (2.5)	1 (0.3)
Religion		
Hinduism	287 (91.1)	303 (96.2)
Buddhism	4 (1.3)	4 (1.3)
Muslim	10 (3.2)	4 (1.3)
Kirat	14 (4.4)	4 (1.3)
Literacy status		
Illiterate	53 (16.8)	42 (13.3)
Primary	101 (32.1)	70 (22.2)
Lower	88 (27.9)	67 (21.3)
Secondary		
Secondary	53 (16.8)	80 (25.4)
Higher		
Secondary and above	20 (6.3)	56 (17.8)
Age category (years)		
Less than 20	20 (6.3 )	35 (11.1)
20 to 30	139 (44.1)	107 (34.0)
30 to 40	102 (32.4)	88 (27.9)
40 to 50	48 (15.2)	54 (17.1)
50 to 60	6 (1.9)	26 (8.3)
60 and above	-	5 (1.6)

Table 2 shows that majority of workers were non-smokers in both the industries [230 (73%) vs. 223 (70.8%)]. Half of the workers in the industries had reported of chewing tobacco [152 (48.3%) vs. 145 (46%)]. Majority of the workers were not using personal protective equipment as mask to protect from dust at their workplaces in both the industries [261 (82.9%) vs. 238 (75.6%)].

**Table 2.** Personal habit of the workers in the jute and textile industry.

Characteristics	Jute Industry (n = 315) n (%)	Textile Industry (n=315) n (%)
**Smoking Status**		
Smoker	65 (20.6)	90 (28.6)
Non Smoker	230 (73.0)	223 (70.8)
Ex-smoker	20 (6.3)	2 (0.6)
Tobacco		
Non Chewer	163 (51.7)	170 (54.0)
Chewer	152 (48.3)	145 (46.0)
Alcohol		
No Intake	203 (64.4)	174 (55.2)
Intake	112 (35.6)	141 (44.8)
Personal		
Protective		
Equipment		
Non Users	261 (82.9)	238 (75.6)
Users	54 (17.1)	77 (24.4)

Most of the workers had the working experience of less than five years in both the industries [134 (42.5%) vs. 180 (57.1%)]. There was no participant with working experience of more than 15 years in the jute industry where as about 36 (11.4%) workers had working experience of 15 to 20 years and 12 (3.8%) workers had working experience of more than 20 years in the textile industry (Table 3).

**Table 3.** Distribution of workers according to their working experience in the industries.

Working Experience (years)	Jute Industry (n=315) n (%)	Textile Industry (n = 315) n (%)
Less than 5	134 (42.5)	180 (57.1)
5 to 10	105 (33.3)	53 (16.8)
10 to 15	76 (24.1)	34 (10.8)
15 to 20	-	36 (11.4)
More than 20	-	12 (3.8)

Table 4 shows the workers with their peak expiratory flow rate less or more than 80% of their predicted one. The proportion of workers having less than 80% of their predicted peak expiratory flow rate was far less in both the industries. About 26 (8.3%) and 23 (7.3%) workers produced the hardest blow to measure peak expiratory flow rate less than 80% of their predicted one in jute and textile industry respectively.

**Table 4.** Categorization of workers with their peak expiratory flow rate (PEFR).

PEFR	Jute Industry (n=315) n (%)	Textile Industry (n=315)
< 80% predicted PEFR	26 (8.3)	23 (7.3)
> 80% predicted PEFR	289 (91.7)	292 (92.7)

Distribution of workers according to respiratory disorders in the jute and textile industry is shown (Table 5). Cough was reported by more than one-fourth of workers in the jute industry while it was reported by less than one-tenth of workers in the textile industry. Upper respiratory disorder was prevalent among more than one fifth of workers in the jute industry while it was prevalent among around one twentieth of workers in the textile industry.

**Table 5.** Respiratory disorders of workers in the jute and textile industry.

Respiratory Disorders	Jute Industry (n=315) n (%)	Textile Industry (n = 315) n ( %)
Cough	86 (27.3)	26 (8.3)
Throat Pain	15 (4.8)	-
Upper	68 (21.6)	18 (5.7)
Respiratory		
Problem		
Difficulty in	16 (5.1)	6 (1.9)
Breathing		
Chest tightness	4 (1.3)	17 (5.4)
Chronic	1 (0.3)	2 (0.6)
Bronchitis		
Cough with	21 (6.7)	5 (1.6)
sputum		
Asthma	-	1 (0.3)
Tuberculosis	-	1 (0.3)

Difficulty in breathing, representing lower respiratory tract symptoms was prevalent among 16 (5.1%) workers in the jute industry while it was prevalent among 6 (1.9%) workers in the textile industry. As much as 4 (1.3%) and 17 (5.4%) workers in the jute and textile industry reported chest tightness as lower respiratory tract problem respectively. Only one worker was diagnosed as chronic bronchitis in the jute industry. Two workers were diagnosed as chronic bronchitis in the textile industry.

Table 6 shows the difference of means of PEFR of workers with respect to type of industry, gender and use of personal protective equipment. Mean score of PEFR of workers in jute mill was lower than the workers of textile industry. Likewise, means of PEFR of workers was higher among males than females; PPE users than non-users at work place.

**Table 6.** Comparison of PEFR of the workers with respect to industry, gender and PPE use.

Characteristics	Category	Mean PEFR	SD
Industry	Jute	466.35	90.461
	Textile	522.73	118.419
Gender	Male	507.47	103.850
	Female	371.67	75.423
PPE use	Yes	486.13	101.586
	No	526.56	129.036

Increase in means of PEFR of workers was seen according to the increase in height, and weight. However, the means of PEFR also increased with age except in category 20-30 years (Table7).

**Table 7.** Comparison of PEFR of all the workers by height, weight and age.

Characteristics	No. of workers	Mean PEFR	SD
Height category (cm) <150	41	381.71	75.990
150-155	62	427.26	80.697
155-160	151	481.92	108.577
160-165	183	508.03	96.837
165-170	136	518.97	91.839
> 170	57	580.70	124.440
Total	630	494.54	109.002
Weight category (kg)			
<45	83	417.83	90.595
45-55	235	484.17	99.878
55-65	199	515.18	103.547
65-75	83	525.06	105.869
>75	30	566.67	142.909
Total	630	494.54	109.002
Age category (years)			
<20	55	501.82	90.351
20-30	246	496.26	114.449
30-40	190	485.05	103.866
40-50	102	491.37	105.070
50-60	32	525.31	131.615
>60	5	558.00	122.760
Total	630	494.54	109.002

## DISCUSSION

Difference in proportion of workers was found between jute and textile industry in terms of gender, marital status, literacy status, smoking status, alcohol intake, age category and body mass index category. Cough (27.3%), upper respiratory problem (21.6%), difficulty in breathing (5.1%), chest tightness (1.3%), chronic bronchitis (0.3%) were found in jute industry while cough (8.3%), upper respiratory problem (5.7%), difficulty in breathing (1.9%), chest tightness (5.4%), chronic bronchitis (1.6%), asthma (0.3%) and tuberculosis (0.3%) were found in textile industry. Byssinosis (2.3%), chronic bronchitis (4.5%) and upper respiratory infections (7.2%) were found among cotton textile workers.^15^ Acute upper respiratory infection (14.2%), chronic bronchitis (0.3%), acute lower respiratory infections (0.77%), asthma (0.4%) were revealed among the workers.^11^ Textile mill workers in Bangladesh were shown to suffer from chronic bronchitis and/or asthma (5.7%) and chest tightness or breathlessness (4.3%). ^6^

Higher respiratory problem among workers (38%) was found in a jute mill.^8^ In another study conducted among 95 workers of a jute mill, chest pain (34.7%), cough with sputum (11.58%), dry cough (7.37%), nasal catarrh (3.16%), breathlessness (2.11%) were major respiratory symptoms.^9^ Less than 80% predicted PEFR was seen among 8.3% workers in jute industry and 7.3% workers in textile industry. Similar findings were revealed by other studies, where PEFR was lower in high dust exposure group in comparison to low dust exposure group.^16,17^ Statistically significant low PEFR was identified among textile workers with symptoms of cough, chronic bronchitis and/or asthma, chest tightness or breathlessness.^18^ Exposure of dust and plant source fine particulate matter among Bengali workers had lower PEFR values than controls.^19^ Studies had related jute dust to respiratory symptoms.^20^ Early respiratory symptoms may be a risk factor for subsequent loss of pulmonary function in cotton textile workers.^2^

Use of personal protected equipment was found to be neglected in both the industry. Use of protective equipment was lower in jute industry than in textile industry. However, the workers in both the industry were at risk of respiratory problems because of the low practice of personal protective equipment as mask and the synergistic effect of smoking habit.

Mean score of PEFR of workers in jute mill was lower than the workers of textile industry. Lower PEFR of workers in jute mill could be due to lower practice of PPE as mask and the level of dust, which was higher in the jute industry than textile industry.

There are some limitations to this study. First, the study cannot be generalized as it has been carried out in one jute industry and one texile industry. Environmental measurement (exposure measurement) for dust was not carried out at the workplaces. This study recommends that appropriate personal protective measures should be used by all the workers in dust producing industries. Examination of workers in the industries should be routinely done to know the health status of the workers.

## CONCLUSIONS

The workers in both the industry were at risk of respiratory problems. Chronic respiratory problems did not appear to be alarming in both the industries. Acute respiratory disorders predominate the chronic one in both the industries. Most of the workers had more than 80% predicted peak expiratory flow rate. Low practice of personal protective equipment such as mask was observed. This study can be useful for the policy makers, industrial managers and researchers to make appropriate plan to improve the health status of the workers in dust producing industries. This study also recommends further investigations in the industries to make definitive inferences about the effect of the risk factors with respiratory symptoms of the workers.
